# Intelligent retrieval method for power grid operation data based on improved SimHash and multi-attribute decision making

**DOI:** 10.1038/s41598-022-25432-7

**Published:** 2022-12-05

**Authors:** Songyan Zhao, Xiaoli Guo, Zhaoyang Qu, Zhengming Zhang, Tong Yu

**Affiliations:** 1 School of Computer Science, Northeast Electric Power University, Jilin, China; 2 Electric Power Research Institute, Guangxi Power Grid Co., Ltd, Nanning, China

**Keywords:** Mathematics and computing, Information technology

## Abstract

IN the trend of energy revolution, power data becomes one of the key elements of the power grid. And an advance power system with "electric power + computing power" as the core has become an inevitable choice. However, the traditional search approach based on directory query is commonly used for power grid operation data in domestic and international. The approach fails to effectively meet the user's need for fast, accurate and personalized retrieval of useful information from the vast amount of power grid data. It seriously affects the real-time availability of data and the efficiency of business-critical analytical decisions. For this reason, an intelligent retrieval approach for power grid operation data based on improved SimHash and multi-attribute decision making is proposed in this paper. This method elaborates the properties of SimHash and multi-attribute decision making algorithms. And an intelligent parallel retrieval algorithm MR-ST based on MapReduce model is designed. Finally, real time grid operation data from multiple sources are analyzed on the cloud platform for example. The experimental results show the effectiveness and precision of the method. Compared with traditional methods, the search accuracy rate, search completion rate and search time are significantly improved. Experiments show that the method can be applied to intelligent retrieval of power grid operation data.

## Introduction

In the trend of energy revolution, power data becomes one of the key elements of the power grid. And an advance power system with "electric power + computing power" as the core has become an inevitable choice. Power grid data has the characteristics of large size, various types, low value density, fast processing speed, etc. In recent years, large amount of data is collected and processed by automation systems and devices such as smart substations, supervisory control and data acquisition (SCADA), wide area measurement systems (WAMS) and smart meters^1^. Currently, various parts of the power system are the source of generating big data on grid. For example, on the generation side, flexible DC as the representative of the new transmission technology, new power equipment, so that the grid to collect more complex structure and different sources of data. On the electricity consumption side, along with the generation of integrated energy supply and vehicle-network convergence, interactive demand response between power companies and customers under Internet aggregation is rapidly developing. As a result, it is possible to obtain more accurate and massive power data than before^2–4^. In the background of the dramatic increase in grid operation data, the traditional retrieval method of catalog query has the disadvantages of few available resources, information overload, low accuracy, low completeness, low efficiency and slow retrieval speed. Traditional methods are no longer sufficient to meet the needs of companies to obtain data efficiently.

After decades of rapid development, the power system has long become a large interconnected system. The system consists of the following components: high-temperature, high-pressure, supercritical and ultra-supercritical units, large-capacity long-distance transmission networks, and real-time changing loads, etc. Data from multiple platforms and sources constitute the big data of electricity, which makes the new power system operation data rise dramatically. This data contains a wealth of value for society and the power system. Analysis and mining of relevant power data helps power systems to quickly retrieve matching information and facilitate advance scientific decision-making. However, the real-time operation data of the power grid has the characteristics of time, localization and accumulation. These characteristics cause the data retrieval methods to have difficulties in retrieval, low retrieval efficiency and other problems. Therefore, how to achieve efficient retrieval of grid operation data in a large-scale data size environment is an important challenge^5^.

To address these issues, this paper dissects algorithms such as SimHash and multi-attribute decision making based on the MapReduce programming model^6^. In this paper, an algorithm MR-ST is proposed for grid operation data with parallel intelligent retrieval. The proposed algorithm extracts feature from the ontology database and maps them to a unique retrieval feature vector. The retrieved vector will be converted to an intelligent search of its feature vector. Finally, example analysis of multi-source real-time grid operation data on Hadoop platform. The experimental results show that.

- The algorithm proposed in this paper can efficiently and accurately retrieve the retrieval results of large amount of grid operation data, and the search accuracy rate, search completion rate and retrieval time are significantly improved compared with traditional methods.
- The algorithm not only effectively solves the problems of intelligent retrieval of large-scale grid operation data and rational utilization of software and hardware resources, but also changes the dependence of traditional methods on high-dimensional data.

The proposed approach is applicable to the intelligent retrieval of grid operation data, and it can effectively improve the efficiency of data acquisition and mining of key business analysis and decision making for each power company in real-time.

## Traditional SimHash algorithm and its improvement

The traditional SimHash algorithm calculates the similarity of the original vector by generating a fingerprint. The more similar the original data, the more similar the fingerprint obtained. This method identifies the degree of difference of each content on the bit^7^. The SimHash algorithm steps are shown below^6^

1. Initialize the vector F (f dimensions) to 0 and the SimHash fingerprint S (f bits) to 0.
2. Extracts the keywords inputed by the user into the retrieved document and calculates their weights.
3. The same Hash function is used to calculate the fingerprint b (f bits) of the keyword, and each bit in b is scanned one by one. If in b, its i-th position is 1, then in F, the value of its i-th position plus the weight of the related keyword. Otherwise, subtract the weight of related keywords.
4. In F, if the value on the i-th position of F is positive, then let the value on the i-th position in S be 1. Otherwise, set the value to 0, and output the fingerprint S.
5. The Hemming distance of the fingerprint S is compared and then compared to the specified threshold. Finally, the comparison result is used to determine whether the vectors are similar or not.

SimHash algorithm has many flaws. For keywords, there is a lower search efficiency and accuracy of the extraction problem and there is also the case of inefficient matching of retrieval. In addition, it also appears that the retrieval accuracy of the weight calculation is not refined. Therefore, for the current problem of feature extraction of power grid operation data, this paper uses NLPIR PARSER Chinese word separation tool for Chinese word separation. The tool is capable of new word discovery, adaptive word splitting and keyword extraction^8^. The resulting keywords $t_{(i,n)}$ are obtained and the speed and accuracy of keyword calculation is improved. The lexical weights $w_{1(i,j)}$ of keywords are calculated by hierarchical analysis, where the weight of nouns is set to 0.3, verbs are set to 0.2, etc.^9^. Furthermore, the traditional word frequency-inverse document frequency TF-IDF algorithm is used to calculate the word frequency weight $w_{2(i,j)}$ of keyword $t_{(i,n)}$^8,10^. On this basis, the algorithm adds new word weights $w_{3(i,j)}$ and word span weights $w_{4(i,j)}$, and finally derives the weights $W_{(i,n)}$ for keyword $t_{(i,n)}$. The relevant definitions are as follows

### Definition 1

Term frequency TF.

TF (Term Frequency) indicates: how often a keyword appears in the search database. Therefore, the definition of keyword frequency TF for keyword A is given by the following equation.

1 $$t{\text{f}}_{i,j} = \frac{{n_{i,j} }}{{\sum\limits_{Q} {n_{i,j} } }}$$

where $n_{i,j}$ is the frequency of occurrence of $t_{(i,n)}$ in the retrieved database $D_{{\text{j}}}$. Denominator $\sum\limits_{Q} {n_{i,j} }$ is the total number of occurrences of all words in the search database $D_{{\text{j}}}$.

### Definition 2

Inverse Document Frequency IDF.

IDF (Inverse Document Frequency) indicates: the rating of the importance of a word as a keyword. Therefore, the definition of the inverse document frequency IDF for a keyword $t_{(i,n)}$ is as follows.

2 $${\text{idf}}_{i} = \lg ({N \mathord{\left/ {\vphantom {N {n_{i,j} }}} \right. \kern-\nulldelimiterspace} {n_{i,j} }} + \alpha )$$

where N is denoted as the total number of words in the database; $n_{i,j}$ is the total number of keywords $t_{(i,n)}$ in the database; $\alpha$ is an empirical value, generally taken as 0.01, 0.1, 1.

### Definition 3

Traditional TF-IDF.

1. Find the word frequency weight of keyword $t_{(i,n)}$ , defined as follows.
  3 $$w_{2(i,j)} { = }tf_{i,j} \times idf_{i} = \frac{{n_{i,j} }}{{\sum\limits_{Q} {n_{i,j} } }} \times \lg \left( {\frac{N}{{n_{i,j} }} + \alpha } \right)$$
2. Find the new word weight $w_{3(i,j)}$ for keyword $t_{(i,n)}$, defined as follows.
  4 $$w_{3(i,j)} = \frac{{\frac{{n_{i,j} }}{{\sum\limits_{Q} {n_{i,j} } }} \times \lg \left( {\frac{N}{{n_{i,j} }} + \alpha } \right) + len(t)}}{{\sqrt {\sum\limits_{p = 1}^{\beta } {\left[\frac{{n_{i,j} }}{{\sum\limits_{Q} {n_{i,j} } }} \times \lg \left( {\frac{N}{{n_{i,j} }} + \alpha } \right)\right]^{2} } } + len(t)}}$$
  where **Len(t)** refers to the length of the new word; $\beta$ represents an empirical value, and its value can be randomly selected. In general, in the appropriate value, any $\beta$ value can be taken by cross-validation with reference to the sample.
3. Find the word span weight $w_{4(i,j)}$ for keyword $t_{(i,n)}$ , defined as follows.
  5 $$w_{4(i,j)} = \frac{{l_{i} }}{L}$$
  where $l_{i}$ refers to the number of paragraphs in which the keyword appears and **L** indicates the total number of paragraphs.

### Definition 4

Improved TF-IDF.

The weight $W_{(i,n)}$ of keyword $t_{(i,n)}$ is defined as follows

6 $$\begin{aligned} W_{{({\text{i}}.j)}} &= w_{{1_{(i.j)} }} \times w_{{2_{(i.j)} }} \times w_{{3_{(i.j)} }} \times w_{{4_{(i.j)} }} \hfill \\ & = w_{{1_{(i.j)} }} \times [\frac{{n_{i,j} }}{{\sum\limits_{Q} {n_{i,j} } }} \times \lg (\frac{N}{{n_{i,j} }} + \alpha )] \times \frac{{\frac{{n_{i,j} }}{{\sum\limits_{Q} {n_{i,j} } }} \times \lg (\frac{N}{{n_{i,j} }} + \alpha ) + len(t)}}{{\sqrt {\sum\limits_{p = 1}^{k} {[\frac{{n_{i,j} }}{{\sum\limits_{Q} {n_{i,j} } }} \times \lg (\frac{N}{{n_{i,j} }} + \alpha )]^{2} } } + len(t)}} \times \frac{{l_{i} }}{L} \hfill \\ \end{aligned}$$

## Multi-attribute decision making algorithm

The multi-attribute decision making algorithm TOPSIS (Technique for Order Preference by Similarity to Ideal Solution) is used to calculate the utility values of grid operation data based on the improved SimHash^11,12^. The method can filter the retrieved grid operation data in a targeted way. The TOPSIS uses the "ideal solution" and "negative ideal solution" of the multi-attribute decision making method to rank them one by one and determine the combined performance of each search solution. If there is a solution which is the closest to the ideal solution and the farthest from the negative ideal solution, it is the optimal solution retrieved among **k** retrieval solutions. Expert knowledge can be embedded into the system in advance to provide objective scoring of grid operation data from various factors (e.g. weather, peak periods, etc.) at different times and environments. The process of comparing and evaluating grid operation data based on the TOPSIS method in the data retrieval process is as follows

1. The method uses grid operation data performance characteristics as indicators for grid data evaluation and retrieval of qualitative requirements to create an initial decision matrix ${\mathbf{VF}}_{k}.$
  1. Similarity retrieval vector ${\mathbf{V}}_{k} = [{\mathbf{v}}_{1} \;\;{\mathbf{v}}_{2} \;\cdots\; {\mathbf{v}}_{k} ]$ of retrieved grid operation data
  2. Set ${\mathbf{B}}_{k} =[{\mathbf{b}}_{1}\;{\mathbf{b}}_{2}\;\;\cdots\;{\mathbf{b}}_{k} ]$ to be a vector of retrieved qualitative demand indicators
  3. The initial matrix can be expressed as ${\mathbf{VF}}_{k} = ({\mathbf{vf}}_{ij} )_{m \times n}$.
  Where ${\mathbf{vf}}_{ij}$ denotes the score value of its $j$ th indicator in the $i$ th data
2. Normalization of the decision matrix
  7 $${\text{vf}}_{ij}^{*} = {{{\text{vf}}_{ij} } \mathord{\left/ {\vphantom {{{\text{vf}}_{ij} } {\sum\limits_{i = 1}^{k} {{\text{vf}}_{ij}^{2} } }}} \right. \kern-\nulldelimiterspace} {\sum\limits_{i = 1}^{k} {{\text{vf}}_{ij}^{2} } }}$$
  The normalized decision matrix is ${\mathbf{VF}}_{k}^{*} = (vf_{ij}^{*} )_{{{\text{m}} \times n}}$.
3. Normalize the retrieved qualitative demand vector ${\mathbf{B}}_{k} = [{\mathbf{b}}_{1}\;{\mathbf{b}}_{2}\; \cdots\;{\mathbf{b}}_{n} ]$. The normalized demand vector is ${\mathbf{B}}_{k}^{*} = [{\mathbf{b}}_{1}^{*} \;{\mathbf{b}}_{2}^{*} \;\cdots\; {\mathbf{b}}_{n}^{*} ]$. where ${\mathbf{b}}_{j}^{*}$ represents the weight of the normalized demand indicator **j**.
  8 $${\text{b}}_{j}^{*} = {{{\text{b}}_{j} } \mathord{\left/ {\vphantom {{{\text{b}}_{j} } {\sum\limits_{j = 1}^{n} {{\text{b}}_{j} } }}} \right. \kern-\nulldelimiterspace} {\sum\limits_{j = 1}^{n} {{\text{b}}_{j} } }}$$
4. Calculate the distance from each retrieved grid operation data $P_{i} = (1 \le {\text{i}} \le k)$ to the ideal solution and the negative ideal solution
  9 $$\varphi_{i}^{ + } = \sqrt {\sum\limits_{j = 1}^{n} {\left[ {{\text{b}}_{j}^{*} \left( {{\text{vf}}_{ij}^{*} - {\text{vf}}_{j\_best}^{*} } \right)} \right]^{2} } }$$
  10 $$\varphi_{i}^{ - } = \sqrt {\sum\limits_{j = 1}^{n} {\left[ {{\text{b}}_{j}^{*} \left( {{\text{vf}}_{ij}^{*} - {\text{vf}}_{j\_worst}^{*} } \right)} \right]^{2} } }$$
  where, $n$ refers to the total retrieval of grid operation data on qualitative characteristics; ${\mathbf{vf}}_{ij}^{*}$ indicates the score obtained after normalizing ${\mathbf{vf}}_{ij}$; ${\mathbf{b}}_{j}^{*}$ represents the demand weight obtained after normalization of ${\mathbf{b}}_{j}$; ${\mathbf{vf}}_{j\_best}^{*}$, ${\mathbf{vf}}_{j\_worst}^{*}$ represent the **j**th subscoring value of the ideal solution and the **j**th subscoring value of the resulting negative ideal solution after normalization, respectively.
5. Calculate the utility value $U_{{\text{i}}}$ for all retrieved grid operation data $P_{i} = (1 \le {\text{i}} \le k)$.
  11 $$U_{i} = \frac{{\varphi_{i}^{ - } }}{{\varphi_{i}^{ + } + \varphi_{i}^{ - } }}$$

## Intelligent retrieval of grid operation data based on MapReduce model

### Principle of MR-ST algorithm

MapReduce is proposed by Google. MapReduce has the practical effect of enabling a parallel programming model for processing massive data sets^13^. The MR-ST algorithm based on improved SimHash and multi-attribute decision techniques explains the traditional SimHash algorithm and combines it with MapReduce model, TF-IDF and multi-attribute decision making algorithms. MR-ST can effectively implement an intelligent retrieval process for parallel retrieval of grid operation data on a cloud platform.

MR-ST algorithm retrieves similar vectors of massive grid operation data in parallel based on MapReduce model. The Map function in the algorithm can autonomously complete the slicing operation of each data. Reduce function is to sort the results of multiple Map. Due to their known performance, they can be used in combination with other functions. Algorithms parallelize the processing of Map and Reduce functions, and a programming model can be formed.

*Introduction to the Map function*

Map function provides simultaneous access to $n$ data slice. The function extracts the retrieved row numbers and their retrieved attribute values in combination with the retrieval requirements and then performs the mapping calculation. This procedure requires a call to the relevant function that generates a < key, value > pair.

*Introduction to the Reduce function*

The Reduce function receives the results from the map of each node and performs the merge calculation. And the functions store datasets in a distributed file system (HDFS).

The MR-ST algorithm intelligent retrieval process is divided into six steps and three retrieval phases.

1. Obtain keyword retrieval vector ${\mathbf{G}}_{K}$, sequence retrieval vector ${\mathbf{X}}_{K}$, retrieval feature vector ${\text{R}}_{{\text{K}}}$.
2. The search preprocessing yields keyword search database ${\mathbf{D}}_{j}$, sequence search database ${\mathbf{D}}_{j + 1}$, search feature database ${\mathbf{D}}_{j + 2}$.
3. Improving SimHash similarity calculation, multi-attribute decision making and comprehensive score calculation.

**Figure 1:**
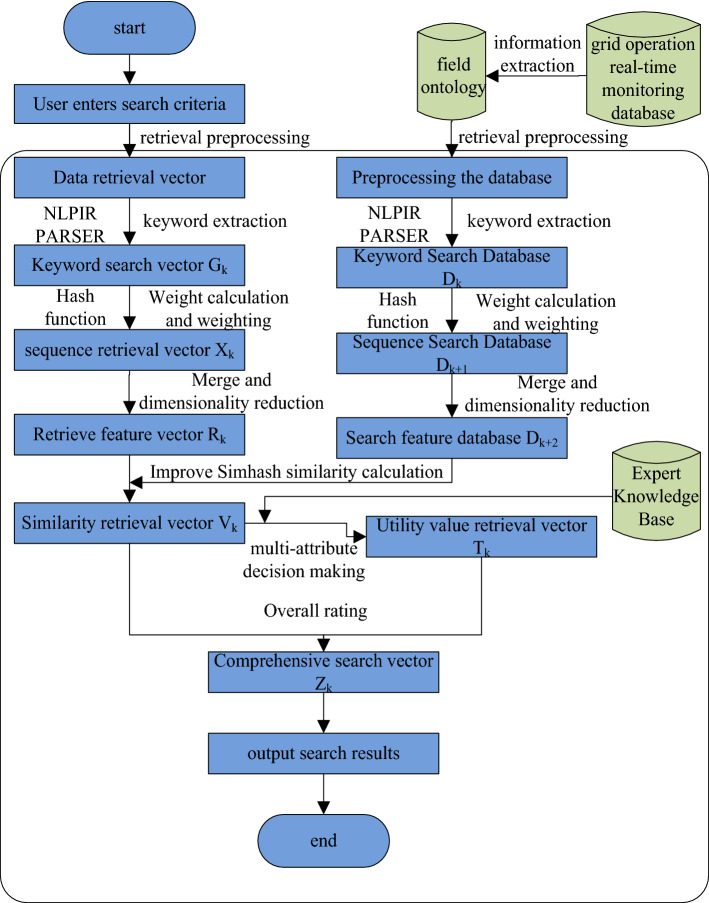
Principle block diagram of MR-ST algorithm.

The principle block diagram of MR-ST algorithm is shown in Figure 1.

### MR-ST algorithm construction

Firstly, the paper sets the parameter symbols in the algorithm.

i. Set the database to ${\mathbf{D}}_{k}$, $K \in R$, and the row number to $ID$. The attribute value of column **j** of row **i** of the database is set to $V_{{\text{i,j}}}$ and $V_{i,j} \in V$. $t_{(i,n)}$ refers to the **n**th keyword in the **i**th row.
ii. Suppose the user inputs the search criteria data table as $Y_{k}$, $K \in R$, and the row number to $id$. The attribute value of column **j** of row **i** of the database is set to $E_{i,j}$ and $E_{i,j} \in E$. $m_{(i,n)}$ refers to the **n**th keyword in the **i**th row.

The MR-ST calculation process is shown below.
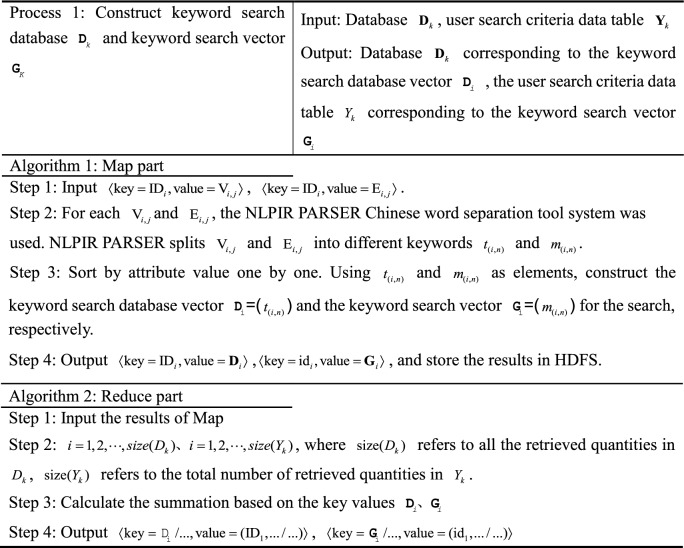


The algorithm stores the output results in HDFS as keyword search database sub vectors ${\mathbf{d}}_{k} = ({\text{ID}}_{i},{\mathbf{D}}_{i} )$, keyword search sub vectors ${\mathbf{g}}_{k} = ({\text{id}}_{i},{\mathbf{G}}_{i} )$. If a ${\text{key}}$ corresponds to multiple values of ${\text{value}}$, then ${\text{value}}$ is split and corresponds to ${\text{key}}$ one by one. For different searches, proximally stored in the keyword search database vector ${\mathbf{d}}_{k}$, keyword search vector ${\mathbf{g}}_{k}$. Searches with the same ${\text{key}}$ in ${\mathbf{d}}_{k},\;{\mathbf{g}}_{k}$ are each clustered into the same vector. Therefore, we get ${\mathbf{D}}_{K} = ({\mathbf{d}}_{k} ),\;{\mathbf{G}}_{{\text{k}}} = ({\mathbf{g}}_{k} )$.
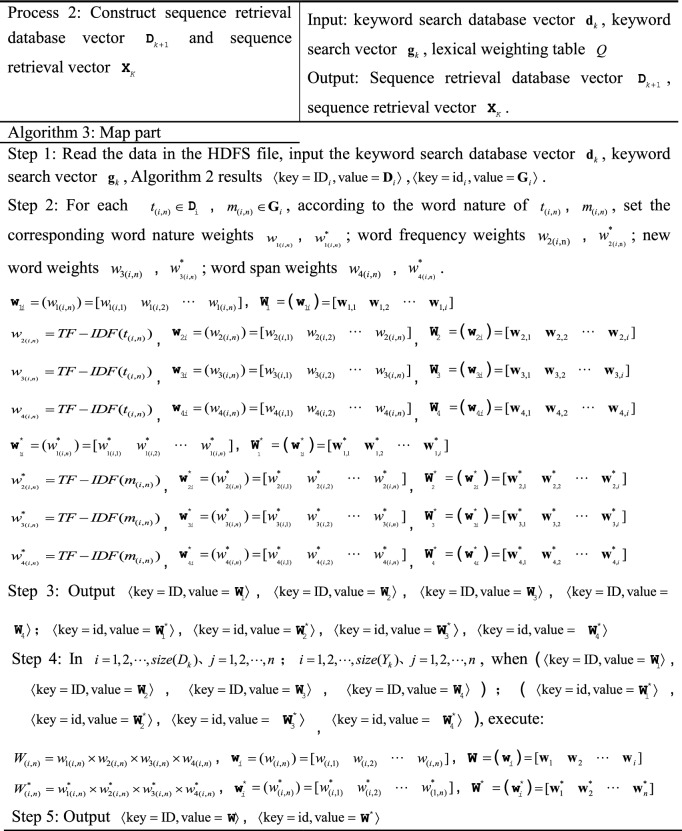

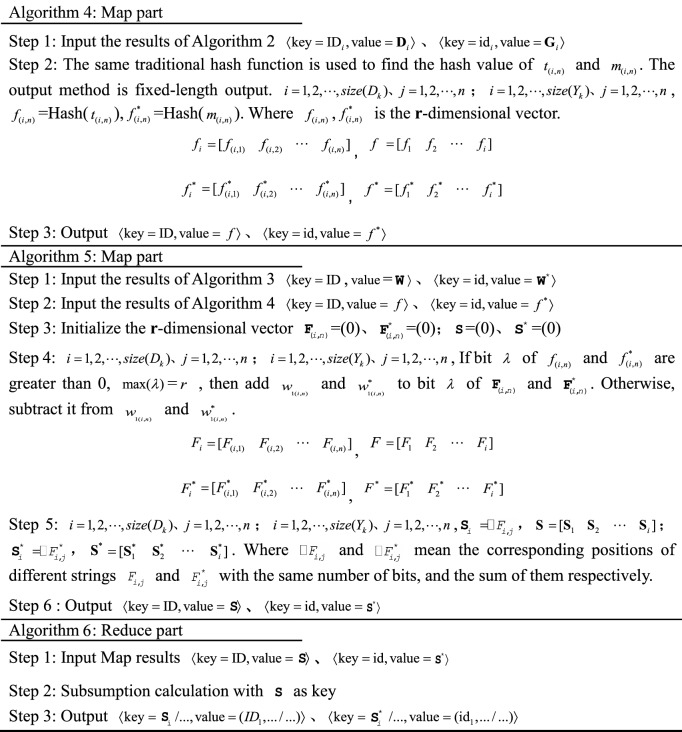


The results are stored in HDFS as sequence retrieval database sub vector ${\mathbf{d}}_{k + 1} = ({\text{ID}}_{i},{\mathbf{S}}_{i}^{{}} )$ and sequence retrieval sub vector ${\mathbf{x}}_{k} = ({\mathbf{ID}}_{i},{\mathbf{S}}_{i}^{*} )$, $D_{k + 1} = [f_{1} \;f_{2} \;\cdots\; f_{i} ]$, ${\mathbf{X}}_{k} = [f_{1}^{*} \;f_{2}^{*} \; \cdots \;\,f_{i}^{*} ]$. If a ${\text{key}}$ corresponds to multiple values of ${\text{value}}$, then ${\text{value}}$ is split and corresponds to ${\text{key}}$ one by one. The value are stored in the sequence search database sub vector ${\mathbf{d}}_{k + 1}$, sequence search sub vector ${\mathbf{x}}_{k}$ in the order of ID, id from largest to smallest, respectively.
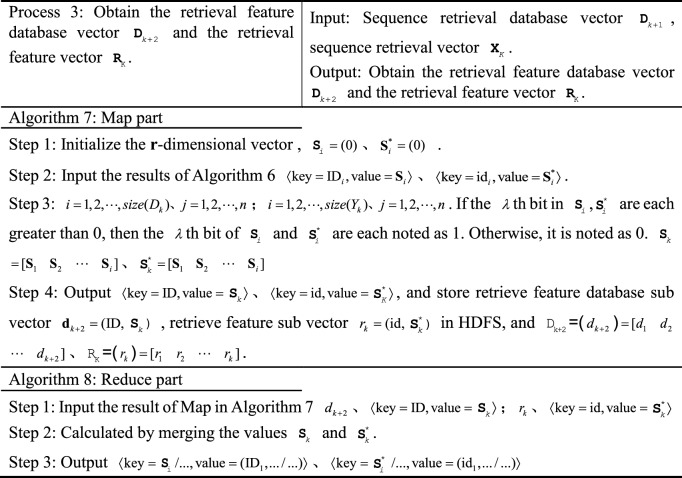


In the output, the similar search vectors ${\mathbf{S}}_{i}$ and ${\mathbf{S}}_{i}^{*}$ are clustered together respectively. According to the number of ${\text{ID}}$ and ${\text{id}}$, the corresponding similar search sub vectors $d_{k + 2}$ and $r_{k}$ are stored in HDFS, and ${\text{D}}_{{\text{k + 2}}} = (d_{k + 2} ) = [d_{1} \;d_{2} \;\cdots \;d_{k + 2} ]$, ${\text{R}}_{{\text{K}}} = (r_{k} ) = [r_{1} \;r_{2} \;\cdots\; r_{k} ]$.
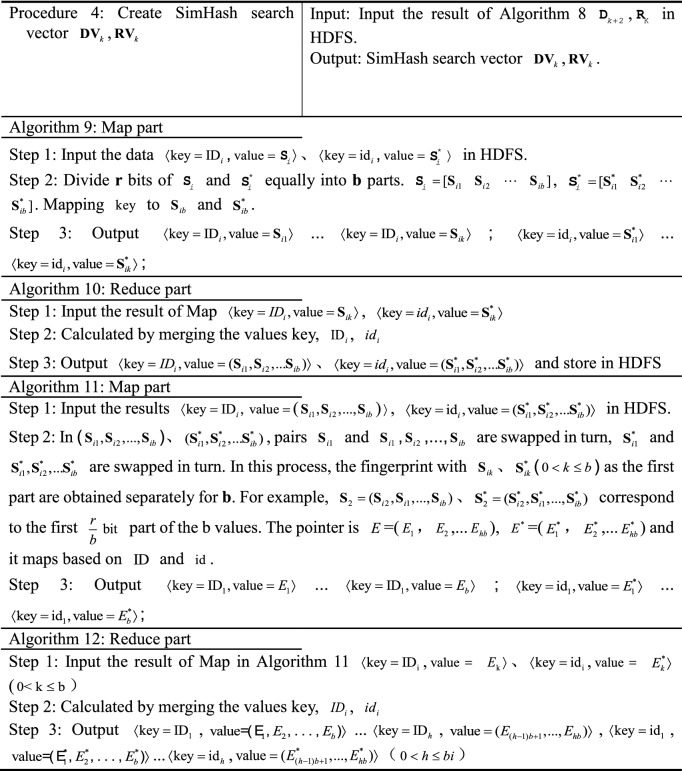


The results are stored in HDFS as a search vector ${\mathbf{DV}}_{k} = (\mathbf{ID}_{i},E_{i} )$,  $E_{i} = (E_{(h - 1)b + 1},...E_{hb})$, ${\mathbf{RV}}_{k} = (\text{id}_{i}, E_{i}^{*} )$, $E_{i}^{*}$ = $(E_{(h - 1)b + 1}^{*},...,E_{hb}^{*} )$. ${\varvec{ID}}_{i}$, $E_{i}$, $S_{i}$; ${\text{id}}_{i}$, $E_{i}^{*}$, $S_{i}^{*}$ correspond to each other respectively.
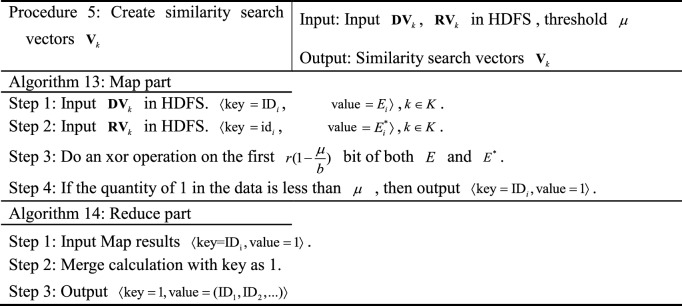


The vectors that can be clustered together in the output are the vectors with similarity. According to ${\text{ID}}$, its corresponding retrieval is stored in HDFS as similarity retrieval sub vector ${\mathbf{v}}_{K}$, and the similarity retrieval vector is ${\mathbf{V}}_{k} = [{\mathbf{v}}_{1} \;{\mathbf{v}}_{2} \;\cdots\;{\mathbf{v}}_{k} ]$.
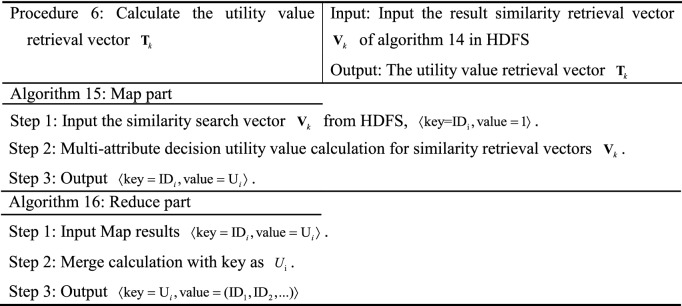


In the output, the utility value retrieval vectors ${\mathbf{T}}_{k}$ are clustered together. According to ${\text{ID}}$, sort according to utility values in turn and store them in HDFS.
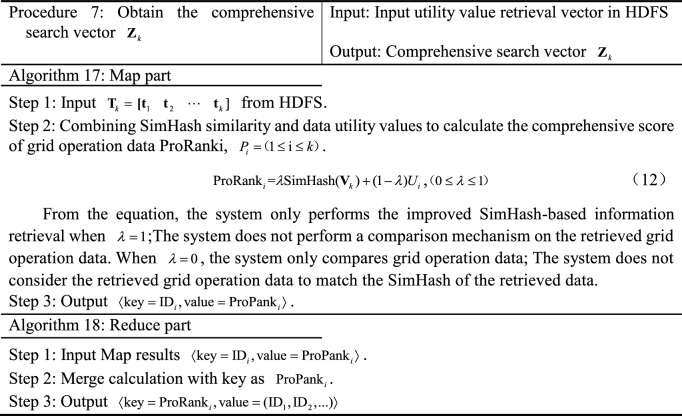


In the output, the comprehensive search vector ${\mathbf{Z}}_{k}$ is clustered together. According to ${\text{ID}}$, the sorting is done sequentially based on the comprehensive score and stored in HDFS and ${\mathbf{Z}}_{k} =[{\mathbf{z}}_{1}\,{\mathbf{z}}_{2}\,\;\cdots\;{\mathbf{z}}_{k} ]$. During the execution of the algorithm, the system retrieves and merges at the same time to improve the efficiency of retrieval. Process 1 to process 3 represents the process of retrieving records. Process 4 and 5 represent the retrieval similarity calculation. Process 6 and 7 are retrieval similarity multi-attribute decision and comprehensive score calculation. The computational complexity of the algorithm MR-ST is the sum of the complexity of all processes. It can be expressed as

13 $$O(|i|(3|n| + |j| + |r|)) + O(|i|(4|b| + |r|(1 - \frac{3}{b})) + O(|i|(|k| + |i|)))$$

## Experiments and results analysis

In this section, the accuracy and completeness of the MR-ST algorithm are verified, and its parallelism and retrieval efficiency are examined. The experimental setup has 16 nodes as follows: 1 management node, 1 IO node, 14 computing nodes. This experiment builds the cluster experiment environment in Hadoop platform. The system is built with Rocky Linux for computing clusters, configured with 11th Gen Intel(R) Core(TM) i5-1135G7@2.40 GHz 2.42 GHz, 16 GB RAM, 500 GB hard disk, and Hadoop-3.3.0. The system uses NLPIR PARSER Chinese word separation tool. The tool can achieve 96.95% accuracy for single machine word splitting and 982 KB/s speed for word splitting. The experiment used the threshold, similarity length as the similarity calculation criteria in the reference^7^. When two vectors’ Hemming distances less than or equal to 3, two vectors are defined to similar. They are called similar vectors and similarity length use 64 bits. For the preprocessing of experimental data is the method in the reference^8^. The operational data of the 3rd quarter of 2021 of a power company in China Southern Power Grid is used as the experimental data for this experiment.

The main indicators to determine the performance of grid operation data retrieval are the search precision rate (PR) and the search recall rate (RE) of the search query. The formulae for calculating the precision and recall of grid operation data are as follows

14 $$PR = \frac{{\sum\limits_{Satisfy} {vector} }}{{\sum\limits_{{A{\text{ll}}}} {vector} }} \times 100\%$$

where, $\sum\limits_{{A{\text{ll}}}} {vector}$ is the total number of grid operation data files retrieved during the search process; $\sum\limits_{Satisfy} {vector}$ is the number of all retrieved grid operation data files that match the retrieval requirements.

15 $$RE = \frac{{\sum\limits_{Retrieve} {vector} }}{{\sum\limits_{System} {vector} }} \times 100\%$$

where, $\sum\limits_{Retrieve} {vector}$ refers to the number of relevant grid operation data files retrieved; $\sum\limits_{System} {vector}$ indicates the number of relevant grid operation data files in the system.

**Figure 2:**
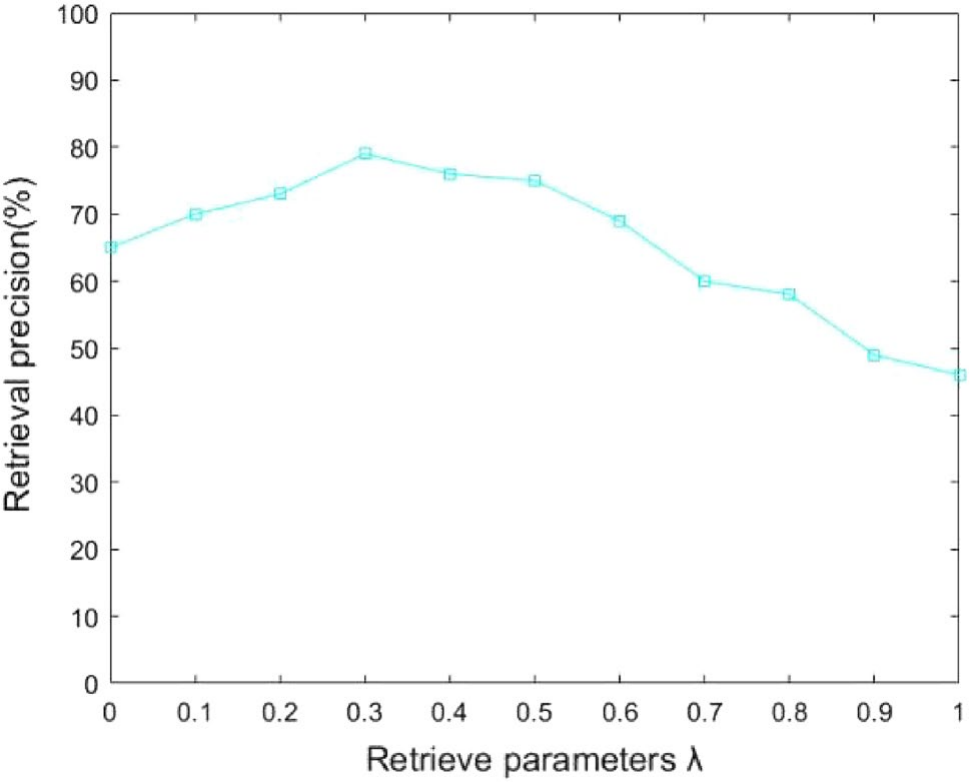
The relationship between parameter $\lambda$ and retrieval precision.

The amount of data retrieved for all grid operations that meet the retrieval requirements can be preset to a threshold value according to Eq. (12). The higher the accuracy rate calculated by the algorithm, the better the performance of grid operation data retrieval. Obviously, the accuracy of the grid operation data and the related ranking of the retrieved results are directly influenced by the value of $\lambda$. After analyzing the experimental results, the relationship between $\lambda$ and the search accuracy is shown in Fig. 2. As can be seen from Fig. 2, when the value of $\lambda$ is equal to 0.3, the impact on the retrieval performance of grid operation data is greater, and the retrieval accuracy rate is optimal. Therefore, the utility value calculation based on the TOPSIS method has the greatest impact on the grid operation data retrieval when the value of $\lambda$ is equal to 0.3.

### Precision, recall and efficiency analysis of the algorithm

To evaluate the precision, recall and efficiency of MR-ST algorithm, the retrieval results of this paper were compared with those of the four retrieval algorithms involved in the reference^14^ and the reference^15^. The compared algorithms are as follows

1. YD is an intelligent commodity information retrieval based on semantic similarity and multi-attribute decision method^14^
2. YZ is an information and knowledge organization intelligent retrieval method based on metadata^15^
3. DQ is multilevel index-driven place name information retrieval method^16^
4. GS is keywords-based temporal information retrieval method over relational databases^17^

The evaluation standard of the accuracy of similarity search can be measured by PR, RE and search time (TI). The search content is set to be fixed, each automatic search is set to be performed at 0.2 s intervals, and the number of iterations is performed 6 times. The PR, RE, TI of the above methods were recorded separately, and the comparison of the results is shown in Table 1.

**Table 1:** Accuracy comparison among four algorithms.

Algorithm	Data source	PR/%	RE/%	TI/s
YD	Company1	79	86	5
YZ	Company1	75	90	3
DQ	Company1	66	58	12
GS	Company1	42	19	7

Similarly, the accuracy of the MR-ST algorithm is verified using a small dataset and the results are compared with those of the SimHash-based retrieval algorithm. The system sets six Map and Reduce processes as experimental tasks; the experimental data contains 12 attributes, the size of the dataset is 200 KB (2500 items) and 6000 KB (25,660 items) respectively, and the detection results of the algorithm are shown in Table 2.

**Table 2:** Detection results of MR-ST and SimHash algorithm.

Algorithm	Data source	Data size	PR/%	RE/%	TI/s
SimHash	Company1	200	38	85	5
MR-ST	Company1	200	96	92	3

The MR-ST algorithm obtained a PR value of 96%, an RE value of 92%, and a TI value of 3/s, so the MR-ST algorithm has good retrieval performance. In addition, the MR-ST algorithm retrieves about 60% more PR, 7.6% more RE, and 40% more TI than the traditional SimHash algorithm. MR-ST algorithm has more accurate and efficient retrieval performance.

**Figure 3:**
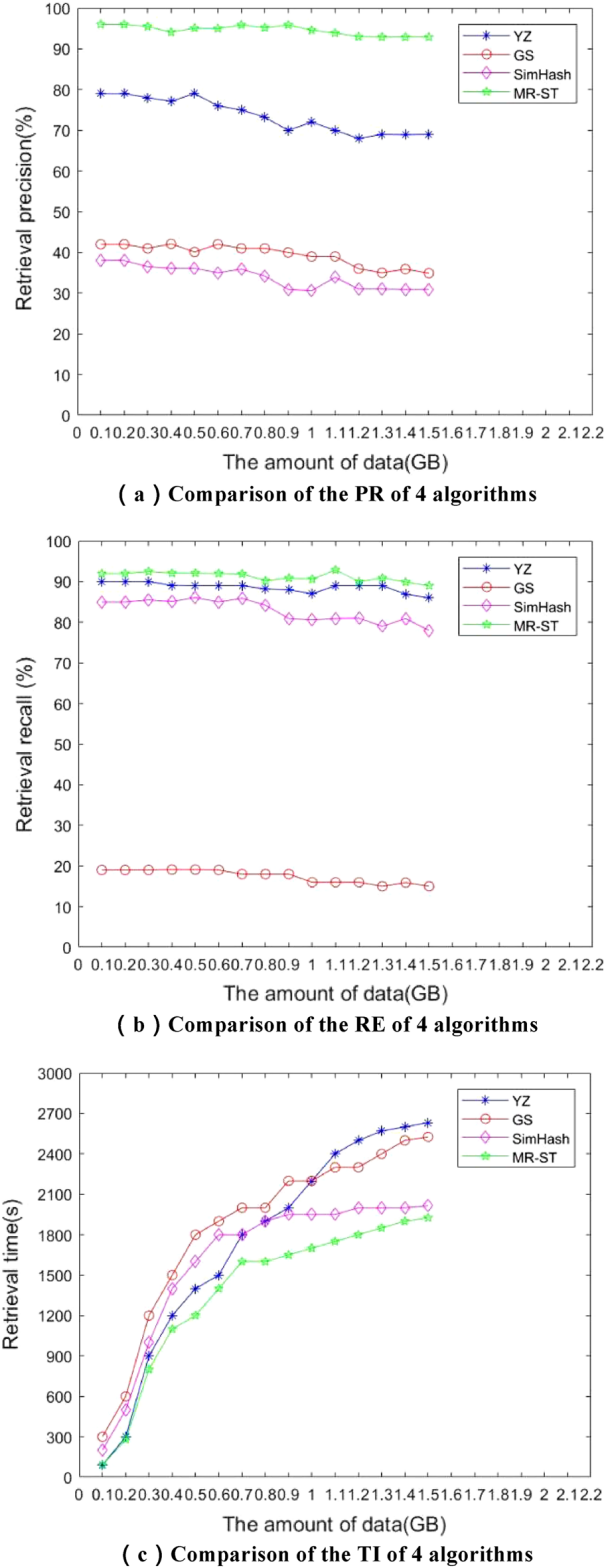
Comparison of retrieval performance of several algorithms.

From Tables 1 and 2, it can be seen that the SimHash algorithm has worse retrieval performance than the MR-ST algorithm, and the MR-ST algorithm has the best retrieval performance. Moreover, the PR, RE, and TI values of MR-ST algorithm are higher than those of other algorithms, so MR-ST algorithm has the optimal retrieval performance. In addition, among the four algorithms in Table 1, the YZ algorithm has the best balance of PR and retrieval time, and its performance is optimal. The GS algorithm has the lowest PR and RE, and also takes relatively long to search, and has the worst balance, so its search performance is the worst. To verify the retrieval performance of the proposed MR-ST algorithm in this paper, a larger dataset is used and compared with the traditional SimHash algorithm, the YZ algorithm with the strongest retrieval performance and the GS algorithm with the worst retrieval performance in Table 1. In the experiment, 12 Map and Reduce task processes are set, and the experimental data comes from the operational data of a power company in China Southern Power Grid. The data size is 1.5 GB, and each record contains 11 attributes. The obtained experimental results are shown in Fig. 3.

From Fig. 3, it is clear that the MR-ST and YZ algorithms have the highest execution efficiency under the same conditions. However, the PR and RE of the MR-ST algorithm are higher than those of the YZ algorithm. Because the MR-ST algorithm directly outputs the similarity retrieval vector during the execution and performs multi-attribute decision making and comprehensive scoring on it. The algorithm simultaneously merges and retrieves similarities. Therefore, MR-ST has short running time and highest efficiency. In addition, compared with the SimHash algorithm, the MR-ST algorithm improves the retrieval efficiency by about 26%, has the highest PR, RE, and TI values, and has the best retrieval performance.

### Influence of parameters on the algorithm

#### Relationship between data size and PR, RE

The experiments were run for 8 MapReduce task processes for data sizes of 2.048 GB, 20.48 GB, and 204.8 GB to verify the effect of data sizes on the PR and RE of MR-ST and SimHash algorithms, and the obtained experimental results are shown in Table 3.

**Table 3:** The impact of the amount of data on the algorithms.

Algorithm	Data source	Data sizes/GB	PR/%	RE/%
SimHash	Company1	2.048	36	81
		20.48	35	83
	Company2	20.48	32	85
		204.8	30	86
MR-ST	Company1	2.048	96	91
		20.48	96	91
	Company2	20.48	95	92
		204.8	95	92

As can be seen from Table 3, data sizes do not affect PR and RE values, but data source does. Because of the differences in the data structure of different data sources, the retrieval performance of the MR-ST algorithm will show a small fluctuation (PR value floats within 2%, RE value floats within 2%). Even so, it is enough to meet the real-time requirement of similarity retrieval of massive grid operation data. Meanwhile, the PR value obtained from SimHash algorithm retrieval fluctuates within 6.6% and RE value fluctuates within 2.5%. Hence, the impact of data size on MR-ST algorithm is smaller and more stable among different data sources, which is more suitable for intelligent retrieval of rapidly growing huge amount of grid operation data.

#### The relationship between data size and algorithm efficiency

The experimental data used for the 8 MapReduce task processes running in the experiment contains 12 attributes. Experimental data sizes are as follows: 10, 30, 60, 100, 300, 500 MB. The results of the retrieval efficiency of the proposed MR-ST algorithm affected by the size of retrieved data are shown in Table 4.

**Table 4:** The relationship between runtime and data size.

Data sizes/MB	Runtime/s
10	156
30	312
60	502
100	935
300	1203
500	1355

From Table 4, it is observed that the larger the data size, the higher the retrieval efficiency of the MR-ST algorithm. When the data size is small, the search time will be significantly longer. But when the data belongs to a large size, the rising trend of retrieval time will slow down. The reason is that the parallelism of MR-ST algorithm can be fully utilized when the data size is large, and the retrieval efficiency can be maximized. MR-ST algorithm can still maintain a certain retrieval efficiency when all parallel tasks are started. The retrieval efficiency of the MR-ST algorithm proposed in this paper is positively correlated with the scale of data and is suitable for intelligent retrieval of massive grid operation data.

#### Speedup and scalability

**Figure 4:**
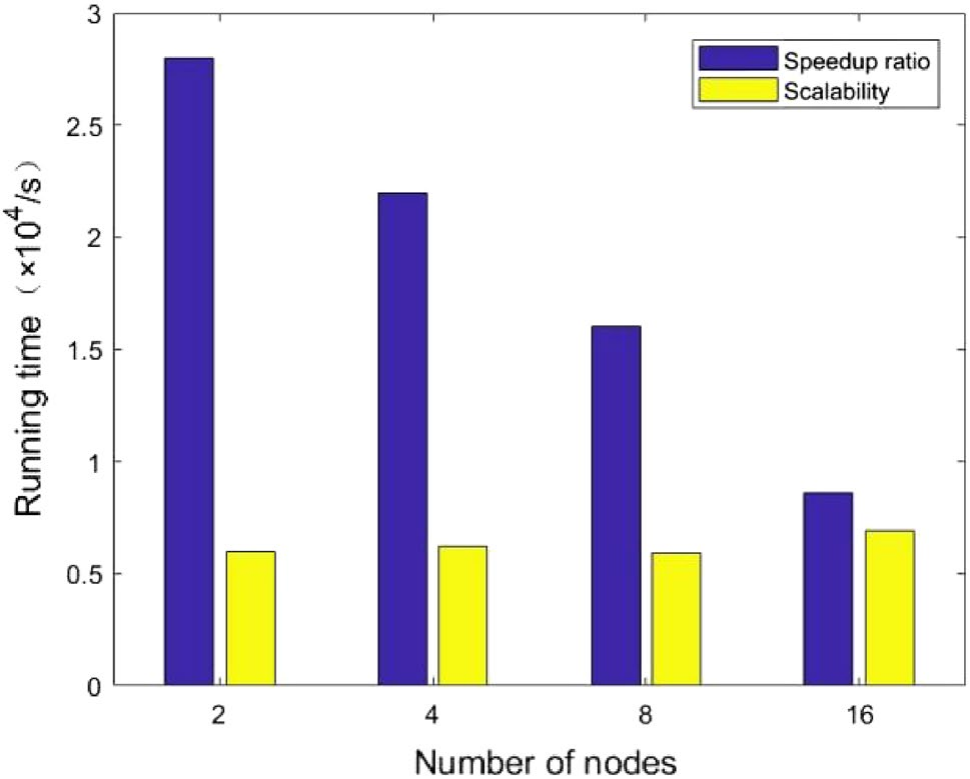
Speedup and scalability of each number of nodes.

The number of nodes is a measure of the system speedup and represents the number of parallel execution processes in the system^6^. speedup refers to the ratio of the time consumed by the same task running in a parallel processor system. It is used to measure the performance and effectiveness of parallel algorithms when the data size is fixed and the number of nodes is increasing. The ideal speedup varies linearly. Scalability refers to improving performance and availability at a larger scale. It indicates the performance of the parallel algorithm when both the number of nodes and the size of the data grow proportionally^7^. For the evaluation of the algorithm speedup, the experiment uses 20 GB of grid operation data volume with grid data size of 2.5, 5, 10, and 20 GB. For the evaluation of the algorithm scalability, experiments were conducted using the number of nodes of 2, 4, 8, 16. The obtained results are shown in Fig. 4.

The proposed MR-ST algorithm has a high speedup ratio and good scalability. However, because of the computer communication overhead and other problems still exist, the acceleration ratio cannot reach the ideal state. The main reason for this is the consumption of computer software and hardware resources, etc. The running time consumption of the algorithm rises slightly when the number of nodes reaches 16. However, the overall execution efficiency of MR-ST algorithm is basically the same, so it has good scalability.

## Conclusion

1. The MR-ST algorithm has a precision rate (PR) > 95% and a recall rate (RE) > 91%. The search precision rate (PR), search recall rate (RE) and search time (TI) of the algorithm are negatively correlated. The algorithm is efficient and stable, and the data size does not affect its PR and RE values, with good retrieval performance and more reliable applicability.
2. The MR-ST algorithm executes efficiently and is positively correlated with data size. The algorithm can perform similarity retrieval with multi-attribute decision and composite score, and can directly output the top similarity vector.
3. MR-ST algorithm has excellent speedup ratio and scalability, suitable for intelligent retrieval of massive grid operation data. Future research will focus on optimization algorithms for massive grid operation data. For example, the impact of a larger number of nodes on the algorithm's retrieval performance. The optimization algorithm will be better applied to the huge amount of grid operation data and can provide data support to enterprises.
4. The proposed approach is applicable to the intelligent retrieval of grid operation data, and it can effectively improve the efficiency of data acquisition and mining of key business analysis and decision making for each power company in real-time.and specifically that, the “Intelligent Retrieval Method for Power Grid Operation Data Based on Improved SimHash and Multi-Attribute Decision Making” has been applied in the scientific research project named "Research on Vulnerability Defense Technology of Power Monitoring System Based on Interdependent Network (047000KK52210031)", which is used for the optimization design of the "data intelligent retrieval module of the massive abnormal data correlation analysis system". The system module has been applied in the field of power grid engineering and has been recognized by the application unit.

## Data Availability

The data that support the findings of this study are available from [Power companies in China] but restrictions apply to the availability of these data, which were used under license for the current study, and so are not publicly available. Data are however available from the authors upon reasonable request and with permission of [Power companies in China].
